# Opium in Hernia

**Published:** 1878-02

**Authors:** 

**Affiliations:** Oxford, Ga.


### OPIUM IN HERNIA.

By A. COUVERT, M.D., Oxford, Ga.

Although for many years a practitioner of medicine in its several branches, I have avoided surgery as much as possible, except in its minor operations, under the belief that no one can attain to much eminence in it outside of a great city, with the facilities there afforded. Nevertheless, I have had to treat some grave cases occasionally, two of which I will briefly report.

Many years ago I was called on by a young physician, a former pupil, to visit a negro boy eight or ten years old, who was suffering from strangulated inguinal hernia. All of the usual measures had been repeatedly and unavailingly tried. Giving a heavy dose of laudanum, I left to prepare my instruments and refresh my memcry for the unaccustomed operation. On my return, after two or three hours, and arranging everthing *secundum artem*, we were about to try taxis again before the knife, when lo! to our surprise and gratification, there was no hernia to reduce. Spontaneous reduction had taken place, and our patient was sleeping profoundly. "Well," said I, "John, we have cured the hernia, but killed the nigger." Not so, however, for he was soon as well as ever.

Some years after the foregoing case occurred, I was called to meet, in consultation, several physicians of experience and reputation, who had under treatment a negro man of about thirty-five, who had been long the subject of inguinal hernia, which recently became strangulated. All usual means were frequently used, and as often failed. Anæsthetics did not then command the attention and confidence that they now do. The knife was decided on, preceded some hours by one grain of morphine, added to large doses given a short time before. When we met to operate two or three hours later, spontaneous reduction had occurred, and a speedy recovery followed.

I think it is an axiom, especially in this department of surgery, that when an operation is necessary, the sooner it is performed the better. I have seen a number of cases of hernia that seemed to call for the knife that were, happily, otherwise relieved. In the foregoing cases, I was within an hour or so of operating, but relief without the knife came within that time. Had I operated, it would have been unnecessary, as the sequel showed. Doubtless there are many cases that require an operation, and many fatal only from delay. Whether to act promptly or delay longer may sometimes be a difficult question for the surgeon to decide.

A distinguished Southern surgeon said, a few years ago, that he had never operated for hernia but once, and the great Liston said in substance that the glory of modern surgery consists more in the avoidance than the performance of operations.

